# Guidelines for managing data and processes in bone and cartilage tissue engineering

**DOI:** 10.1186/1471-2105-15-S1-S14

**Published:** 2014-01-10

**Authors:** Federica Viti, Silvia Scaglione, Alessandro Orro, Luciano Milanesi

**Affiliations:** 1 Istituto di Tecnologie Biomediche, Consiglio Nazionale delle Ricerche, Via Fratelli Cervi 93, 20090 Segrate (Mi), Italy; 2 Istituto di Elettronica e di Ingegneria dell'Informazione e delle Telecomunicazioni, Consiglio Nazionale delle Ricerche, Via De Marini 6, 16149 Genova, Italy

## Abstract

**Background:**

In the last decades, a wide number of researchers/clinicians involved in tissue engineering field published several works about the possibility to induce a tissue regeneration guided by the use of biomaterials. To this aim, different scaffolds have been proposed, and their effectiveness tested through *in vitro *and/or *in vivo *experiments. In this context, integration and meta-analysis approaches are gaining importance for analyses and reuse of data as, for example, those concerning the bone and cartilage biomarkers, the biomolecular factors intervening in cell differentiation and growth, the morphology and the biomechanical performance of a neo-formed tissue, and, in general, the scaffolds' ability to promote tissue regeneration. Therefore standards and ontologies are becoming crucial, to provide a unifying knowledge framework for annotating data and supporting the semantic integration and the unambiguous interpretation of novel experimental results.

**Results:**

In this paper a conceptual framework has been designed for bone/cartilage tissue engineering domain, by now completely lacking standardized methods. A set of guidelines has been provided, defining the minimum information set necessary for describing an experimental study involved in bone and cartilage regenerative medicine field. In addition, a Bone/Cartilage Tissue Engineering Ontology (BCTEO) has been developed to provide a representation of the domain's concepts, specifically oriented to cells, and chemical composition, morphology, physical characterization of biomaterials involved in bone/cartilage tissue engineering research.

**Conclusions:**

Considering that tissue engineering is a discipline that traverses different semantic fields and employs many data types, the proposed instruments represent a first attempt to standardize the domain knowledge and can provide a suitable means to integrate data across the field.

## Background

Several tissue engineering approaches have been proposed in the last years with the aim to mimic the natural processes of tissue regeneration (such as bone and cartilage), by delivering progenitor cells capable of differentiating into osteoblasts and chondrocytes inductive molecules and bioactive three-dimensional scaffolds supporting cellular colonization and matrix deposition [1].

In the last decades, a wide number of *in vitro *and *in vivo *experiments have been carried out in order to test new scaffolds able to guide the process of *ex vivo *connective tissue formation. Experimental preclinical models showed promising results of high scientific interest. However, this approach is far from being considered optimal and mechanisms of tissue formation/remodelling are still not completely elucidated. In particular the cell differentiation process has not been clarified yet, as well as the scaffold features that can positively or negatively influence the cellular fate and, finally, the tissue growth [1]. The identification of biological processes that take place in tissue-engineered constructs could in fact help to further understand the observed mechanism of tissue regeneration. The obtained information can also be useful for improvements in scaffold design through a reverse engineering approach [2].

The complexity of these interdisciplinary studies is even increased by the generation of various types of data at various level of detail, and by the lack of concept models and software infrastructure helping data management and exploitation. In particular, technical details about experiments reported in literature are often scarce and barely accessible to point out the functional effectiveness of tested materials. Systematic reviews and meta-analyses approaches are often based on public scientific literature, thus requiring concepts definition and standardization.

Informatics tools are necessary to organize, and favour accessibility, integration, interoperability and sharing of data, which regards multiple and diverse scientific aspects, such as the presence and/or quantification of bone and cartilage biomarkers, the biomolecular factors intervening in cell differentiation and growth, the morphology and the biomechanical performance of a neo-formed tissue, and, in general, the scaffolds' ability to promote tissue regeneration. In this work a conceptual framework is presented for bone and cartilage tissue engineering domain: authors have defined practical guidelines to drive a structured, reliable and complete method for publishing and sharing (in journals' papers and web based resources) data coming from tissue engineering experiments. Moreover, an ontology, defined as a set of representational primitives suitable to model a domain of knowledge [3], has been developed, to provide a hierarchically structured recognized vocabulary for data explanation, classification and query. The overall framework has been validated with the help of an end user, to guarantee its effectiveness and usability for the tissue engineering community. The Bone/Cartilage Tissue Engineering Ontology (BCTEO) is available at http://bioportal.bioontology.org/ontologies/BCTEO.

### Related work

Data standardization is a rapidly growing area of research, especially in life sciences domain. It represents the basis for data exchange, queries support, publication of reusable knowledge bases and interoperability facilitated across multiple and heterogeneous systems and databases. Standards for structuring data related to the biological field have been proposed, to treat different types of data. In particular, guidelines have been suggested for many aspects of molecular biology defining the minimum set of information (metadata) needed to unambiguously describe experiments and data (for reproduction and interpretation). Major examples of this approach are presented in Table 1.

**Table 1:** Examples of standards suggesting minimum sets of metadata.

**MiMix **[21]	Minimum Information required for reporting a Molecular Interaction experiment
It relies on PSI-MI [22] controlled vocabularies for terms, and its purpose is providing a checklist of the information to be supplied when describing experimental molecular interaction data. It is a module developed within the framework of the MIAPE guidelines.	
**MIAPE **[23]	**Minimum Information About a Proteomics Experiment**
It provides guidance modules for reporting the use of proteomics techniques such as gel electrophoresis and mass spectrometry, and has been developed and proposed to encourage collection and integration of these kinds of data.	

**MIAME **[24]	**Minimum Information About a Microarray Experiment**
It is used to submit fully compliant datasets, to enable the interpretation of the experimental results unambiguously and, potentially, to reproduce the study.	

**MIRIAM Registry **[25]	**Minimum Information Required in the Annotation of Models**
Created for defining the meta-information needed to ensure the re-usability of computational models of biological processes. It aims to maintain, unambiguously and perennially, the identifiers regarding the biomedical domain. The registry retrieves the identifiers in the form of URIs, and provides the http://www.Identifiers.org resolver online service, for their generation.	

Standards have been provided for different fields of molecular biology, reporting the minimum set of information needed to report experiments details and data, in order to enable data reproducibility and interpretation.

For what concerns ontologies, biomedical research area is rich of structured vocabularies and tools to manage them. Ontologies have been developed for genes, proteins, tissues, cells, chemical compounds, anatomy etc. The most used facilities for ontologies consulting and screening are OboFundry [4], NCBO BioPortal [5], OLS [6] and OntoBee [7], which collect many of the existing ontologies in obo and owl formats and provide access to them through searching and browsing facilities.

Nevertheless, no standardization approaches have been developed for tissue engineering. The only semantic instruments provided in this field at the moment seem to be: (1) for what concerns bone domain, the Bone Dysplasia Ontology [8], a comprehensive and formal representation of the skeletal dysplasia domain and related genotypes and phenotypes; and the EVENT-INOH pathway ontology (IEV) [9], used to annotate biological processes, pathways and sub-pathways, where some terms related to growth and tissue regeneration appear; (2) for what concerns material subject, the Nanoparticle Ontology (NPO) [10], describing concepts from the field of cancer nanomaterials.

## Methods

### Tissue engineering context

Adult stem cells demonstrated their multipotency leading to the formation of cartilage, bone, muscle, connective tissue or fat [11].

Autologous stem cells are routinely used in conventional tissue engineering ex vivo in combination with biomaterials to create a cell-based scaffold for replacing or improving the tissue regeneration in vivo. Briefly, cells are typically harvested from donor tissues, isolated and expanded in culture and then associated with biomaterials both of synthetic and biological origin. The cell-material construct may be either cultured in vitro or implanted in vivo, using selectively an ectopic or orthotropic animal model.

**Figure 1:**
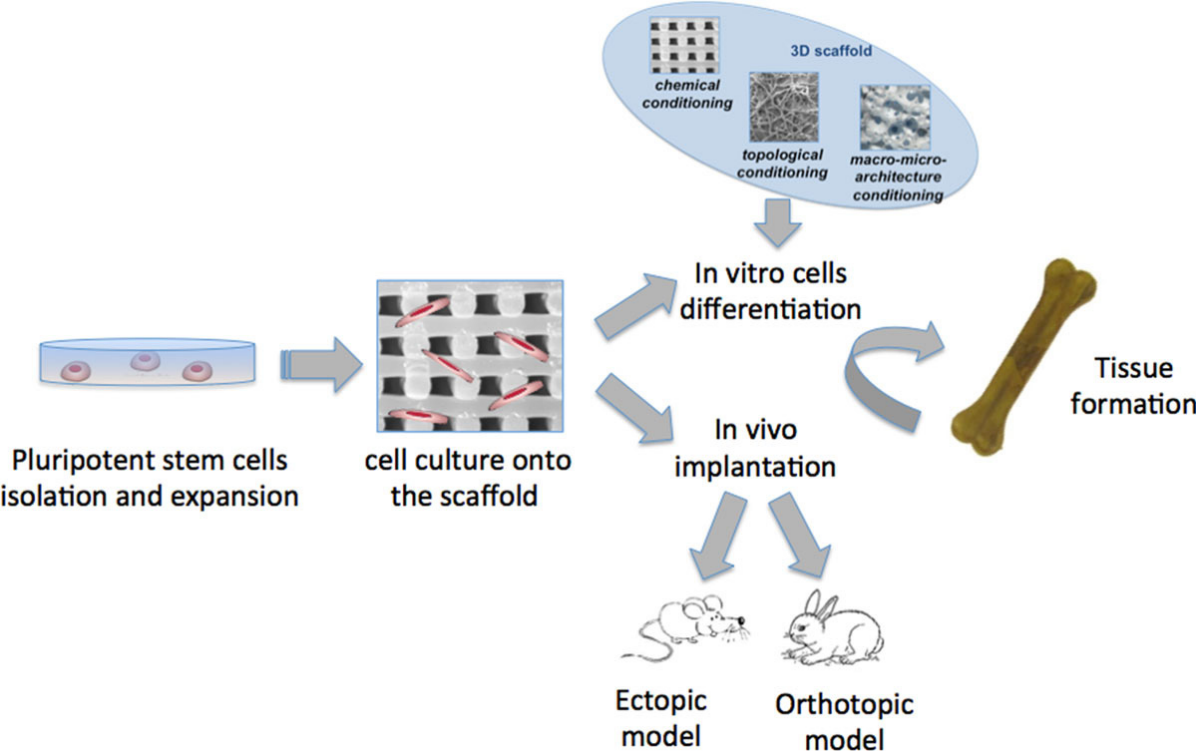
**Simplified schema of the bone/cartilage tissue engineering approach**. Cells extracted from a donor tissue are expanded and seeded in a biomaterial. Tissue growth (and cell differentiation, if starting from stem cells) can be evaluated either in vitro or in vivo. In the latter case, implant can be in ectopic or orthotopic mode, depending on the size of the model organism and the necessity to evaluate the biomechanical stress on the tissue.

In this context, biomaterials have to provide informative microenvironments: material intrinsic nature and structure will anyway transmit a signal that has to be decoded by the colonizing cells and converted into active cellular response. A simplified schema of bone/cartilage tissue engineering approach is shown in Figure 1.

### Ontology approach

The list of minimum information to explain experiments about connective tissue growth on substitute materials has been identified by means of the expertise that authors gained in tissue engineering and in data management and integration. Relying on this experience, literature was manually overviewed for identifying recurrent concepts in papers. A primary list was then enriched with additional information that is often lacking in many published contributions but that appears basic for a correct and complete data report.

On the base of the identified guidelines, which schematically point out macro-areas of the domain, BCTEO has been designed. An ontology is the formal specification of a conceptualization of domain, which consists of a common vocabulary and logical structure providing knowledge framework for annotation, semantic integration, knowledge-based searching and unambiguous interpretation of data [12]. It provides a hierarchical formal representation of a set of concepts through the description of: (1) individuals, which are the basic objects, (2) classes, that are the categories which objects belong to, (3) attributes, which are the features of the objects, and (4) relations, that are the ways objects can be related one another.

BCTEO was developed with the OBO-Edit software in the OBO format [13], the most common format for ontologies in biomedical research field. It is a textual format that attempts to guarantee human readability, ease of parsing, extensibility and minimal redundancy by relying on a list of "stanza". A stanza consists of a name in square brackets (i.e. [Term] for classes or [Typedef] for relations), followed by a series of new-line separated "tag:value" pairs.

To improve the process of ontology creation, authors followed the steps provided by Noy and McGuinness [14] and consisting in: clearly defining the domain and the aim of the project; reusing the existing ontological terms; identifying main terms of the ontology; defining hierarchies and relationships among terms; avoiding inconsistencies. For ontology definition, a top-down approach has been used, by manually screening literature to infer main concepts, and then by increasing the level of detail introducing further in-depth specifications.

For what concerns ontology validation, two aspects have been considered: structure and concepts validation. Structure validation, that regards the consistency of the relations among terms, is guaranteed by the exploitation of the OBO-Edit internal "reasoner", a tool that automatically helps to identify formal errors in the ontology. Concepts validation, which is related to the evaluation of the correctness of the terms inserted in the ontology and their definitions, has been assured by authors' expertise in tissue engineering and strengthened by supervision of skilled end-users (see Tools validation section below).

### Ontological cross-reference

Due to the multidisciplinarity of tissue engineering field, many terms crucial for BCTEO were found already classified and defined in other ontologies. The identification of cross-references among ontologies was aimed to avoid redundancy and terms duplication, to simplify vocabularies exploitation and link among partially overlapping domains. Structured vocabularies containing useful concepts for BCTEO development, and intervening in it, are described in Table 2.

**Table 2:** Structured vocabularies useful for BCTEO

Name	Acronym	Definition	**Ref**.
Brenda Tissue Ontology	BTO	For the source of an enzyme: it comprises terms for tissues, cell lines, cell types and cell cultures from uni- and multi-cellular organisms.	[26]
Common Anatomy Reference Ontology	CARO	For facilitating the interoperability between existing anatomy ontologies of different species, and providing a template for building new anatomy ontologies.	[27]
Chemical Entities of Biological Interest	CHEBI	For chemical compounds of biological relevance.	[28]
Cell Type	CL	For cell types.	[29]
eagle-i research Resource Ontology	ERO	For instruments, protocols, reagents, animal models and biospecimens.	[30]
Gene Ontology	GO	For the annotation of gene products with respect to their molecular function, cellular component, and biological role.	[31]
Protein-protein interaction	MI	For the annotation of experiments concerned with protein-protein interactions.	[32]
Measurement Method Ontology	MMO	For representing the variety of methods used to make qualitative and quantitative clinical and phenotype measurements both in the clinic and with model organisms.	[33]
Microarray and Gene Expression Data Ontology	MO	Concepts, definitions, terms, and resources for standardized description of a microarray experiment	[34]
Medical Subject Headings (MeSH)	MSH	Terms from National Library of Medicine.	[35]
NCI Thesaurus	NCI Thesaurus	For clinical care, translational and basic research, and public information and administrative activities.	[36]
Ontology for Biomedical Investigations	OBI	For investigations: protocols and instrumentation, material, data generated and types of analysis performed on it.	[37]
Phenotype and Trait Ontology	PATO	For phenotypes. Examples of qualities are red, ectopic, high temperature, fused, small, edematous and arrested.	[38]
Physician Data Query	PDQ	Wide range of cancer topics, a listing of some 30,000 cancer clinical trials from around the world, a directory of genetics services professionals, the NCI Dictionary of Cancer Terms, and the NCI Drug Dictionary.	[39]
Systematized Nomenclature Of Medicine Clinical Terms	SNOMEDCT	For medical terms that are used internationally for recording clinical information. They are coded in computer processable mode.	[40]
NCBI organismal classification	TAXON	Taxonomic classification of living organisms and associated artifacts.	[41]

All of them are available for browsing in the NCBO BioPortal web site.

Terms already included in other ontologies have been treated as follows: (1) the value of their namespace has been inherited from the already existing ontology; (2) the term definition is retrieved from the existing one; (3) the term identifier of the already existing ontology is recorded in the 'xref' field, in order to indicate term reuse.

## Results

The value of biological data and the wish to reuse information to infer novel knowledge brought to increase data sharing and exchange in various biological fields. Other than influencing the creation of always more efficient storage infrastructures, this led to demand for standards and vocabularies suitable to enable data compatibility and comparability. Currently, the tissue engineering research field is completely unsupplied with informatics instruments to homogenize information and promote meta-analysis. For filling this lack, (1) an information model, for domain structuring, and (2) an ontology, for domain description, have been developed for bone/cartilage tissue engineering applications.

### The conceptual framework

Authors' experience in tissue engineering enabled generating a critical view about publications in this research field. Commonly, provided data and metadata do not rely on standardized information sharing schemas, limiting possibilities of data exchange, replication and reuse. To answer the need for standardization, a conceptual framework has been proposed, which provides a structure for communicating observations and improving the quality of the contents exposed in a scientific paper. This would facilitate the comprehension of the exposed concepts by readers, the information extraction and the classification of knowledge. Data generated in tissue engineering field are usually complex, due to the high number of variables intervening in the study, and would be meaningless unless a defined standard states which types of data are needed to support and verify conclusions. The identified macro-areas are: (1) 'Material': the identity of the scaffold material and its properties; (2) 'Cell': the type of used cells and the donor; (3) 'In vitro experiments': cells treatment before seeding including quantities, times and growth factors addition; (4) 'In vivo experiments': host and implant model; (5) 'Results': outcome description, consisting in the evaluation and report of the matrix deposition/tissue generation, the material resorption/degradation or adverse effects, such as cytotoxycity; (6) 'Tissue/matrix characterization': morphology, mechanical, biomolecular and biochemical tests to characterize the neo-formed tissue/matrix and to obtain a qualitative and quantitative evaluation of the scaffold performances.

On this basis, the set of minimum information needed to communicate experiments' setup and results has been developed, and detailed in Table 3. The most important aspect of the provided framework is the results explanation. Many techniques exist for assessing it: (1) tissue/matrix structure is commonly evaluated through histology, scanning electron microscopy (SEM), transmission electron microscopy (TEM) or micro-computed tomography (MicroCT) technologies; (2) biomechanical tissue/matrix performances are usually tested through atomic force microscopy (AFM) or dynamic mechanical analysis (DMA) approaches; (3) biomolecular/biochemical aspects can be achieved using histochemistry to highlight content of calcium and glycosaminoglycans in tissue/matrix, or real-time reverse-transcription polymerase chain reaction (real time RT-PCR) and immune-based approaches like immunohistochemistry (IHC) or immunofluorescence (IHF) for detecting biomolecules. The last techniques can target specific, a priori determined, genes and/or proteins, known to be involved in bone (i.e. RUNX2, BSP, ALP, OPN, OC, ON, COL1A1) or cartilage (i.e. COL1A1, COL2A1, COL10A1, ACAN, SOX9) formation. Recently high-throughput techniques such as gene expression microarray and mass spectrometry are beginning to be exploited even in this research field, with the aim of obtaining the whole expression profile and helping the characterization of the biomolecular cellular response [15]. This approach is valuable especially when working with human stem cells, because it allows the determination of the biochemical factors involved in cell differentiation and tissue growth, according to the features of the considered artificial grafts and the provided cues.

**Table 3:** The designed conceptual framework

Sections	Features	Details
**Material **	Material	Type of support material for tissue regeneration exploited in the described experiment.
	
	Material group	Cluster of biomaterials the selected one belongs to: metals, polymers, ceramics, gels, composites, or other.
	
	Features	Morphological and chemico-physical characteristics of the material: bulk properties (total porosity, pore shape and size, pore size distribution, pores volume, pore interconnection); surface properties (chemical/physical functionalization); scaffold properties (sample size and shape *[optional]*); mechanical properties (such as Bulk modulus and maximum strain) *[optional]*; chemico-physical properties (such as resorbability and thermoplasticity) *[optional]*.

**Cell **	Cells type	Type of cells seeded on the biomaterial, both specifying: the type of cells (for example fibroblasts, osteoblasts, chondrocytes); if they are cell lines (reporting the specific cell line code) or primary cells; in this case, the related extraction protocol.
	
	Donor organism	Species of organism the cells derive from.
	
	Donor features	Age, gender, weight and health status of the donor.

***In vitro *experiment**	Cell expansion phase	Time or number of expansions (passages) the cells underwent in plate before being seeded in the biomaterial; type of culture medium used during cell expansion; list of the growth factors that have been added to the cell expansion; for each growth factor, the concentration (mg/ml or Mol) must be reported, together with its isoform, if it exists (as in the case of the Transforming Growth Factor-beta isoforms, which play critical roles in growth regulation and development), and the organism species the growth factor comes from.
	
	Cell culture phase onto the biomaterial	Number (cell quantity) or concentration (number of cells per unit of volume) of cells seeded in the biomaterial; cell seeding efficiency; type of culture medium used during cell culture onto the material; cell culture time; list of the growth factors that have been added to the cell culture onto the biomaterial; for each growth factor, the concentration (mg/ml or Mol) must be reported, together with its isoform, if it exists (as in the case of the Transforming Growth Factor-beta isoforms, which play critical roles in growth regulation and development), and the organism species the growth factor comes from.
	
	Cell characterization	Phenotypic/genotypic characterization of cells before their seeding onto biomaterials: evaluation of the expression of cell surface markers through flow cytometry analysis. *[optional] *

***In vivo *experiment**	Host features	Species of the host, age *[optional]*, gender, weight and health status of the host.
	
	Implant features	Orthotopic (implant placed in the original, correct site) or ectopic (implant inserted under skin); time the biomaterial remains within the animal model; dimension of the implanted samples *[optional]*; surgical procedure for the implant.

**Results**	*In vitro *matrix deposition	Evaluation and report of the matrix deposition in *in vitro *experiments, through morphology, mechanical, biomolecular and biochemical tests (see tests listed below), to obtain a qualitative and quantitative evaluation of scaffold performances. All the results have to be reported specifying the technique used, the applied protocol, the value and measurement unit of the result (in case of quantitative analysis) or the discussion of the result (in case of qualitative analysis).
	
	*In vivo *tissue generation	Evaluation and report of the growth of tissue in *in vivo *experiments, through morphology, mechanical, biomolecular and biochemical tests (see tests listed below), to obtain a qualitative and quantitative evaluation of scaffold performances. All the results have to be reported specifying the technique used, the applied protocol, the value and measurement unit of the result (in case of quantitative analysis) or the discussion of the result (in case of qualitative analysis).
	
	Biomaterial degradation/resorption	Kinetics, amount or percentage of biomaterial degradation/resorption over time, either *in vitro *or *in vivo [optional]*.
	
	Adverse effects	Eventual biomaterial toxicity, evaluated either in vitro (reporting cytotoxicity tests) or in vivo (such as inflammatory response, foreign body reaction, release of degradation by-products)

**Tissue/matrix characterization**	Morphology tests	Results of histological and microscopy tests (i.e. SEM, TEM) used to study structures of the generated tissue/matrix (at level of tissue and cell).
	
	Mechanical tests	Values of main indexes (such as Young module) to define mechanical goodness of the generated tissue/matrix.
	
	Biomolecular tests	Genes and proteins level within tissue/matrix cells. Used technique (typically real time RT-PCR or immunohistochemistry; recently high-throughput technologies such as gene expression microarray were introduced). Examples of bone and cartilage markers are type I collagen, RUNX2 protein, osteonectin, osteopontin, osteocalcin, SOX9.
	
	Biochemical tests	Amount of calcium and mineralization (for bone), amount of aggrecan and glycosaminoglycan (for cartilage); the exploited technique.

Features are grouped by 'concepts', and detailed listing mandatory and non-compulsory (flagged as [*optional*]) aspects to be reported for describing a bone/cartilage experiment.

### The connective tissue engineering ontology

In order to provide roadmaps for data structuring in connective tissue engineering field, a finer informatics tool has been developed: BCTEO. Ontologies represent a source of standardized and recognised descriptive terms: a term belonging to a controlled vocabulary is more precise in the meaning in respect to free-text, since it presents a well established, commonly accepted definition, and a set of synonyms. Furthermore, an ontology is characterized by the relationships among terms, that help for data classification and annotation, semantic based information search (allowing efficient queries), and even inference of new relations among data.

The core of BCTEO conceptually refers to the guidelines proposed in the previous section and it is organized into five main classes: Biomaterial, Experiments, Organism, Tissue, Cellular response. Classes interact at various levels to model the knowledge about bone and cartilage substitute materials.

**Figure 2:**
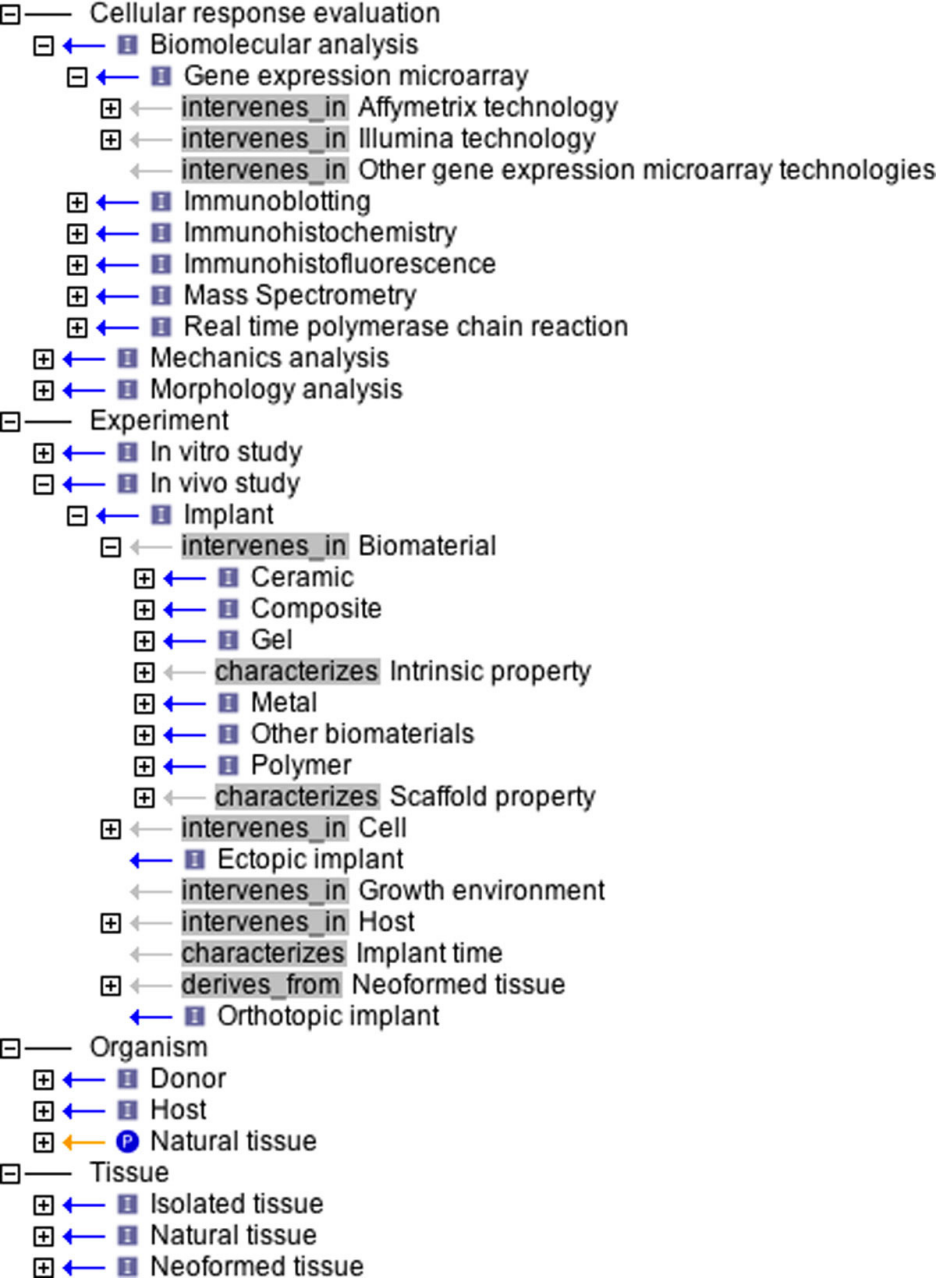
**Partial hierarchical overview of BCTEO ontology**. It was created with OBO-Edit software. A relation, represented as an arrow, links each term to its parent. Exploited relations are: is_a (I), characterizes, intervenes_in, part_of (P), derives_from.

For correctly describing the interactions among ontology classes, new relations have been defined. Additionally to Relation Ontology (RO) [16] terms (is_a, part_of, derives_from), BCTEO relies on two novel relations: 'characterizes' and 'intervenes_in'. The 'characterizes' relation is a general mechanism to represent named attributes. An attribute-like term is related to an ontology term in order to claim its qualities. At best of authors' knowledge no relation exists to link qualities to a term of the ontology. 'characterizes' formal definition is: A characterizes C if and only if: given any c that instantiates C (at time t for continuant), there is a that instantiates A (at time t) and a *characterizes* c, where *characterizes* is an instance-level characterization relation. An example of its usage in BCTEO is: Intrinsic properties characterizes Biomaterial. The 'intervenes_in' relation represents the link between a term that plays a role in a process and the term that describes the process. In RO relations exist that could define similar concepts (for example participates_in), but they are oriented exclusively to model the biological context. 'intervenes_in' is intended to be general purpose, and its formal definition is: given a process P that brings from state S1 (at time t1) to state S2 (at time t2>t1), R intervenes in P if R is a requirement of P; in other words, an instance p of P can exist if and only if an instance r of R exists in the interval (t1, t2). An example of its usage in BCTEO is: Growth environment intervenes_in Cell expansion. Moreover, in OBO format ontology terms have optional fields such as definition, synonyms, comments, or cross-references that can provide additional information to the term name and its relation with parents and children. A partial overview of the BCTEO is shown in Figure 2.

**Figure 3:**
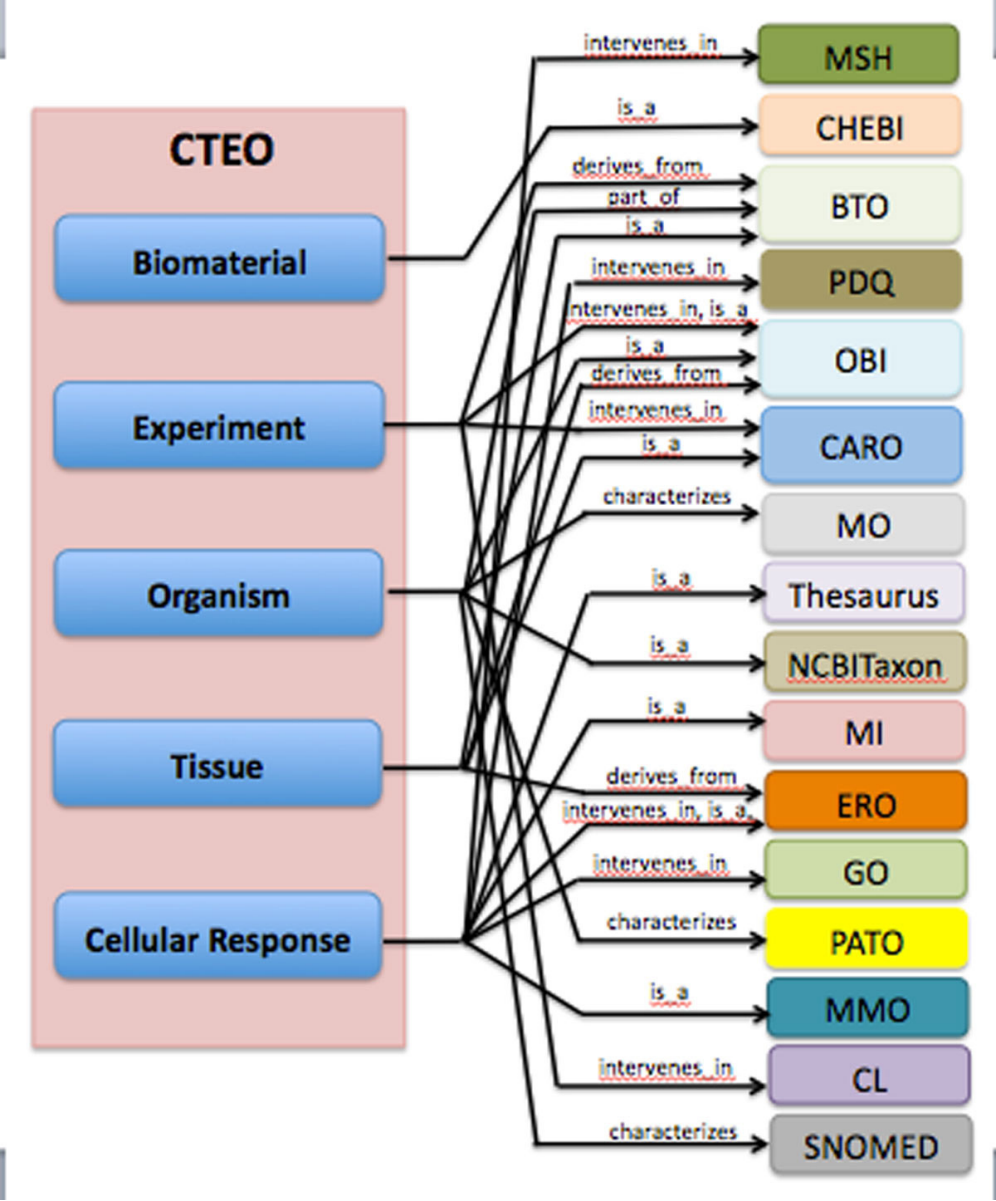
**Relation between BCTEO and other biomedical ontologies**. Domain main concepts (Biomaterial, Experiment, Organism, Tissue, Cellular Response) are shown associated to the external ontologies through the evidenced relations.

To avoid the ex-novo development of concepts previously described in other ontologies, terms already defined are here reported, together with the citation of the related cross-reference. While for the concerns of biological concepts like tissues, cells, cell features, cell types etc. many already defined terms were available in CL, BTO, NCBITaxon and other biology oriented ontologies (for example, 'Real time polymerase chain reaction' is defined in OBI, presenting 0000893 identifier; 'Chondrocyte' belongs to BTO with 0000249 identifier), little contributions were provided by existing structured vocabularies to describe and define investigations on the in vitro and in vivo outcomes. As emerges from Figure 3, where BCTEO relations with external vocabularies and ontologies are pointed out, almost nothing was found in available ontologies concerning biomaterials. This is consistent with the necessity for a concept organization in tissue engineering domain: actually the aim of BCTEO is filling the gap between the biological field, that is a well defined domain, and the material science aspect, that is poor of semantic instruments.

Biomaterials have been classified on the basis of their chemical composition into 'ceramics', 'polymers', 'metals', 'gels' and 'composites'. Materials are even characterized by specific features, which define their behaviour considering two perspectives: (1) the intrinsic properties describe chemical and physical aspects and can be associated directly to each material; (2) the scaffold-related properties depend on the size, the shape and the surface of the graft and are clustered into mechanical and morphological aspects.

### Tools validation

BCTEO and the proposed guidelines have been validated through the consensus of experts. A survey has been designed and distributed to 12 skilled tissue engineers from all over the world (see Table 4 for major details about panel composition). A questionnaire has been organized in four sections, showing the main concepts of BCTEO and guidelines: biomaterial, in-vitro analysis, in-vivo analysis, and cellular response. For each area of interest a set of crucial aspects has been identified and proposed to the experts, asking them to provide a score of relevance (1='absolutely not necessary', 2='not necessary', 3='optional', 4='necessary', 5= 'absolutely necessary').

**Table 4:** Experts' panel composition

Country	Males	Females
Italy	3	2

Portugal	0	1

Spain	0	1

Switzerland	3	0

United States	2	0

**Total no of experts**	**8**	**4**

Gender and provenance characterization of the panel of tissue engineers chosen to comment and validate the developed tools.

**Figure 4:**
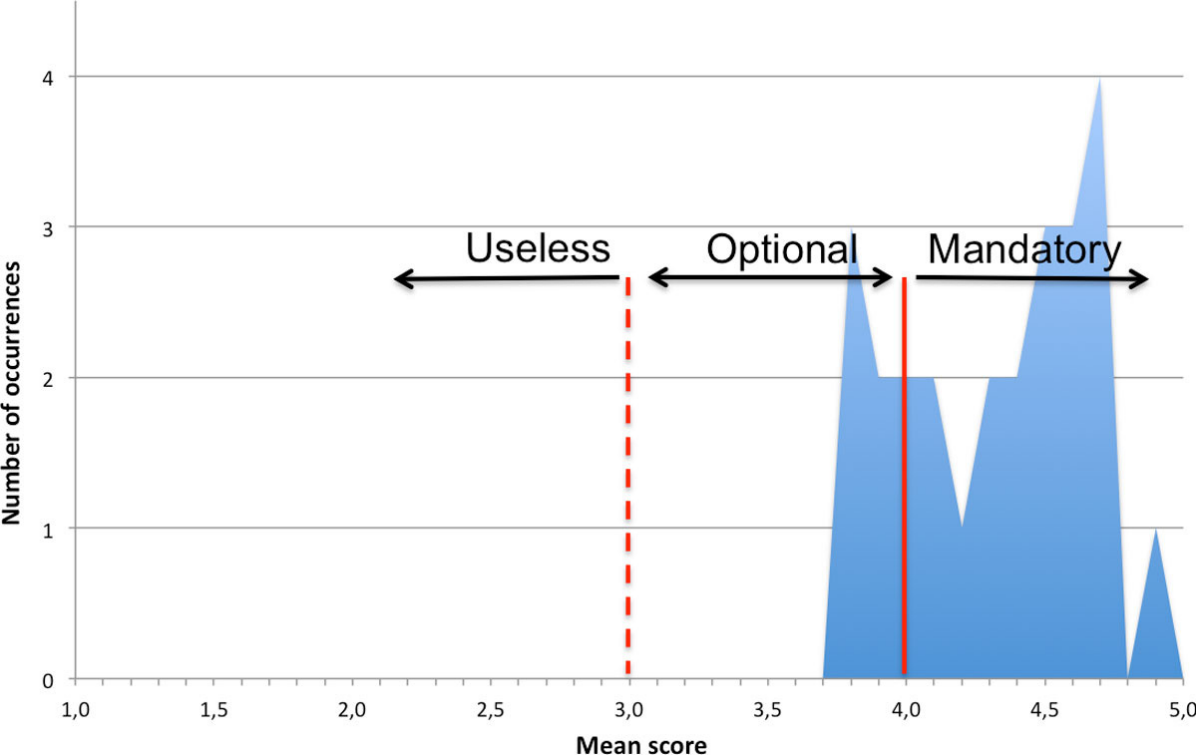
**Distribution of the mean scores obtained by survey's questions**. The whole set of questions obtained a mean score widely upper than the sufficiency (represented by score 3.0). On the basis of the mean score different guidelines' aspects are flagged as optional (to distinguish to the other, that are mandatory by default). None of the proposed aspects has been recognized as useless.

For each concept, the mean score obtained from all the contacted scientists has been calculated, enabling a relevance comparison (Figure 4). 80% of the concepts obtained a score between 5.0 (maximum mean score) and 4.0 (solid vertical line in Figure 4), confirming that the experts consider as widely necessary those aspects, in order to thoroughly describe a tissue engineering experiment. Other features (20% of the total number of proposed concepts) obtained a partial consensus among the experts (showing a mean score between 3.9 and 3.0 (the minimum value that defines a positive relevance of the concept), thus asserting the interest for these concepts, but not their absolute relevance. These features have been included in BTCEO, and flagged as recommended but not compulsory in the guidelines. No features obtained a score lower than 3.0 (the minimum mean score obtained was 3.8), thus meaning that all the proposed concepts have been considered useful for clearly describing a tissue engineering experiment. An interesting feedback of the survey is represented by the additional suggestions provided by the experts: proposed extra-features (such as the biomaterial cytotoxicity, the surgical technique used for the implant or the cell seeding efficiency) have been added in BTCEO and in the guidelines, thus enriching the provided tools with the community contribution.

## Discussion

Several researches are targeted to the design and realization of biomaterial scaffolds guiding cells to tissue growth. Sharing and collecting experimental data about different biomaterials and diverse experimental conditions could enable data correlation and comparisons, finally supporting the formulation of new hypotheses about the existing materials or the generation of novel intelligent engineered scaffolds. Nevertheless, computational technologies that catalyse discovery by integrating tissue engineering knowledge are few and far from being widely exploited. In this perspective, authors developed two resources helping data sharing and reuse.

Theoretical guidelines have been defined to support information and data structuring in tissue engineering. Reporting data by maintaining adherence to the proposed guidelines will result in clearer scientific publications, and will promote the results' interpretability and comprehension. Currently, the experimental outcome is often presented in qualitative terms. For this reason, a whole section of the guidelines has been dedicated to results description. In fact, numerical quantification is a crucial aspect in all scientific fields, since the improvement of clearness in data exposition (by providing numerical results) would enable their better interpretation, automatic collection and aggregated analysis, thus easily achieving the inference of scaffolds' performance and their comparison. Unfortunately, few analysis techniques report results quantification on numerical basis, while a widely exploited approach for results presentation consists in showing figures reporting images (i.e. for what concerns histology data) or diagrams (such as histograms showing genes or proteins expression under different conditions). Therefore, results evaluation and comparison are often performed visually, obtaining not quantifiable data. For example, histological data showing the potential of scaffolds to support newly formed tissue formation is always reported as a stained image, although it should be shown as a numerical value, besides the histological section. To this aim, in the last years image processing approaches have been published, addressed to quantify the newly formed tissue and/or the matrix deposited within the implant, through segmentation algorithms for either histological [17-19] or x-ray [20] images. Other than helping to move towards more reliable comparative analysis, the effort to provide structured information could support the rapid, systematic capture of bone/cartilage regenerative data and their maintenance in databases, thereby improving access to valuable information.

In addition to the guidelines an ontology has been proposed, whose terms mainly map on information and concepts suggested in the roadmap sections. BCTEO represents the first instrument for semantic approach in tissue engineering field: among other major applications, this ontology can be used as the standardized semantic layer when developing structured collection of data in this domain. Relying on ontologies, database technologies can, for example, promote data comparison and enable faster and more effective queries: characterized by a hierarchical structure, a query on the term 'metal' will retrieve even children terms such as 'stainless steel' and 'titanium', that would be excluded from the results not considering the semantic approach. Another interesting aspect of BCTEO concerns its devotion to a multidisciplinary scientific field that results in the integration of the biological perspective (for what concerns the experimental steps), with the materials science details (for describing the chemical/physical features of the developed scaffolds) and the measurement methods (regarding different techniques to test the neo-formed tissue): this generates a wide presence of cross-references among BCTEO terms and definitions from external ontologies.

Both the developed tools have been validated through a survey proposed to a panel of expert tissue engineers. Some consideration emerges from resulting data. Interestingly, all experts involved in the validation assigned the highest scores to concepts belonging to the "cellular response" section, thus highlighting the importance the scientific community feels about reporting complete details concerning this experimental aspect. Surprisingly, in fact details about molecular, biochemical, mechanical analysis after cell-biomaterial interaction are often missing or incomplete; moreover, only in few cases results are shown as numerical values in scientific papers.

Another interesting observation related to the end-users validation was the disagreement about the features that describe the "cell" section: although experts claim to appreciate a wide number of details about cell source and expansion/culture protocols, they often disagree on the type of required information. This outcome reflects the scientific literature scenario, where experimental data are typically reported in heterogeneous, not standardized mode. In the here presented tools a wide number of features have been considered, in order to integrate different needs and finally obtain a complete set of mandatory features.

## Conclusions

Regulation of bone and cartilage tissue formation and remodelling is one of the main targets of tissue engineering research field. Lots of experiments have been performed in this context, testing the effectiveness of novel substitute materials that promote tissue regeneration. Although widely investigated, it remains a not completely elucidated research field, due to its multidisciplinarity and the complexity of the factors intervening in the experiments. To help scientists in highlighting advantages and disadvantages of biomaterials a theoretical roadmap for experiments description and an ontology for semantic approaches have been designed. Both act in the perspective of information standardization and data sharing, aiming to facilitate data comprehension, helping information classification, recognition and annotation, promoting comparison between works to better evaluate materials response, and hypothesising novel graft features and compositions. The overall platform has been validated by means of the end-user perspective.

Considering that tissue engineering is a discipline that concerns many scientific aspects, traverses many scales (micro-macro levels), and employs many data types, BCTEO represents a first attempt to standardize this variegated domain knowledge, and can provide a suitable mean to integrate data across the field. In particular, database technology can be usefully integrated with an ontology layer, thus improving data integration and simplifying information searching.

Finally, the framework described in this paper has been applied in the context of bone and cartilage tissue engineering: it represents one of the most widely investigated fields of regenerative medicine, due to the constant increasing of articular-skeletal diseases/trauma, mostly in young and sportive people. However, tissue engineering is not restricted to bone and cartilage aspects, and they just represent a starting point to develop informatics tools for regenerative medicine. Therefore, it is worth noting that the here presented tools (guidelines and ontology) can be generalized, thus becoming a useful instrument for semantic approach in the entire tissue engineering field.

## List of abbreviations used

AFM: Atomic Force Microscopy; BCTEO: Bone/Cartilage Tissue Engineering Ontology; BTO: Brenda Tissue Ontology; CARO: Common Anatomy Reference Ontology; CHEBI: Chemical Entities of Biological Interest; CL: Cell Type; DMA: Dynamic Mechanical Analysis; ERO: eagle-i research Resource Ontology; GO: Gene Ontology; IEV: EVENT-INOH pathway ontology; IHC: ImmunoHistoChemistry; IHF: ImmunoHistoFluorescence; MI: Protein-protein interaction; MIAME: Minimum Information About a Microarray Experiment; MIAPE: Minimum Information About a Proteomics Experiment; MicroCT: Micro-Computed Tomography; MIMIx: Minimum Information required for reporting a Molecular Interaction experiment; MIRIAM Registry: Minimum Information Required in the Annotation of Models; MMO: Measurement Method Ontology; MO: Microarray and Gene Expression Data Ontology; MSH: Medical Subject Headings (MeSH); NCBI: National Center for Biotechnology Information; NCBO: National Center for Biomedical Ontology; NCI: National Cancer Institute; NPO: Nanoparticle Ontology; OBI: Ontology for Biomedical Investigations; OBO: Open Biological and Biomedical Ontologies; OLS: Ontology Lookup Service; PATO: Phenotype and Trait Ontology; PCR: Polymerase Chain Reaction; PDQ: Physician Data Query; RO: Relation Ontology; SEM: Scanning Electron Microscopy; SNOMEDCT: Systematized Nomenclature Of Medicine Clinical Terms; TEM: Transmission Electron Microscopy.

## Competing interests

The authors declare that they have no competing interests.

## Authors' contributions

FV designed the ontology and wrote the paper; SS was involved in drafting the manuscript, contributed to ontology generation and developed the survey; AO contributed in BCTEO development and paper revision; LM coordinated the project and maintained the bioinformatics resources. All authors read and approved the final manuscript.

## Declarations

This article is published as part of a supplement. Publication has been funded by the Italian Ministry of Education and Research (MIUR) through Flagship "InterOmics" (PB05) project.

This article has been published as part of *BMC Bioinformatics *Volume 15 Supplement 1, 2014: Integrated Bio-Search: Selected Works from the 12th International Workshop on Network Tools and Applications in Biology (NETTAB 2012). The full contents of the supplement are available online at http://www.biomedcentral.com/bmcbioinformatics/supplements/15/S1.
